# A Dividing Line Impossible?

**Published:** 1921-01-29

**Authors:** W. Keith

**Affiliations:** Beckenham


*To the Editor of* The Hospital.

### A Dividing Line Impossible?

*To the Editor of* The Hospital.

Sir,—The ordinary person (myself, that is to say) takes an interest in the medical service only when he has some personal experience which sets him thinking. Two recent experiences of mine have startled me as to the fallibility of the ordinary general practitioner. He is a splendid fellow and mostly right, but when he goes wrong somebody suffers. My own feeling is that to have the general practitioner placed in a national system may add

**THE EDITOR'S LETTER-BOX,—(*continued*).**

another safeguard against his mistakes. It may by that very officialdom which he so much abhors make him more business-like and more efficient. He may become more conscious of an authority to which he is responsible, and when he unfortunately has to interfere in my affairs I may benefit from the fact that I am not merely dealing with a private individual, but with a member of a system to which he owes responsibility and to which he can appeal for help. This seems to me to be the only answer to the question as to where the dividing line is to be drawn between people in the range of the medical service and those who are to have entire freedom of choice. I don't mind being in a system if everybody else is, but while one man is out I want to be that man. Any other system seems to me open to every charge of class distinction and snobbism, and to make painfully clear what we like to hide as far as possible, that the rich man can get his life saved when a poor man cannot. W. Keith.

Beckenham.

